# Investigation of the effective atomic number dependency on kinetic energy using collision stopping powers for electrons, protons, alpha, and carbon particles

**DOI:** 10.1038/s41598-023-30491-5

**Published:** 2023-03-02

**Authors:** Peiman Rezaeian, Sedigheh Kashian, Rojin Mehrara

**Affiliations:** 1 Radiation Application Research School, Nuclear Science and Technology Research Institute (NSTRI), Tehran, Iran; 2 Physics Department, K. N. Toosi University of Technology, Tehran, Iran

**Keywords:** Applied physics, Nuclear physics

## Abstract

As an important component in medical applications, dosimetry, and radiotherapy studies, the effective atomic number of body tissue, tissue equivalent substances, and dosimetry compounds are investigated. In this research, considering the Coulomb interaction of charged particles, using the collision stopping power and the NIST library data, the effective atomic number of various materials at different energies is calculated for common radiotherapy particles such as electron, proton, alpha, and carbon ions. Taking into account the direct calculation method based on the collision stopping power, the effective atomic number for electron, proton, alpha, and carbon particles is determined for a group of dosimetry and tissue equivalent materials. Results of the calculations based on the collision stopping power showed that in low kinetic energy, the values of the effective atomic number are equal to the total number of electrons in each molecule of the compound, which is quite justified by the physics of Bethe's formulas.

## Introduction

The effective atomic number can be applied to characterize the radiological properties of materials. In the different fields such as radiotherapy and dosimetry the radiological properties of materials can be applied in estimating absorbed dose, buildup factor, and shield design. One of the most effective parameters that define the radiation features of a material is the atomic number. In the event of photons in specific energy, the value of the delivered energy is calculated using the mass absorption coefficient^1^. This parameter depends on the medium atomic number. Also, the amount of the transferred energy of the charged particles is calculated using their stopping power. Thus, in known energies, this parameter varies depending on the medium's atomic number^2^. When the medium is a compound, the atomic number will be defined as the effective atomic number^3^. There have been many studies for calculating the effective atomic number for various materials. Most of these calculations were based on averaging and interpolating the available data^4,5^.

Toward calculating Z_eff_, the charged particle stopping power in a specific medium was carried out of the NIST database. The effective atomic number (Z_eff_) attributes to the target medium and the beam particles. Moreover, it gives information on the radiation interaction characteristics in a specific energy range. In fact, the intrinsic atoms can be substituted for a given molecule with exactly the same number of compatible atoms^2^. The replaced molecules are considered to contain a Z_eff_ number of electrons. Besides, the electron density is related to the interaction zones and correspondingly relates to the effective atomic number, which must be regarded during opting materials and dosimeters^6^.

Consequently, the most prominent characteristics that describe the interaction of radiation with different materials are effective atomic number (Z_eff_) and electron density (N_e_)^7^. In addition, these parameters are used to distinguish materials with the purpose that the most accurate results are obtained during a radiotherapy process. In other words, the most precise diagnostics only can be obtained when the materials are properly individualized^8^. Earlier methods for calculating Z_eff_ were based on percent composition by mass of elements, mass attenuation coefficient, Auto Z_eff_ code, and stopping power tables^9–12^.

The percent composition by mass of elements is not dependent on type and energy. While the mass attenuation coefficient calculations, which can be used only when determining the Z_eff_ for photon, depend on energy. This refers to the physical concept that in various energies of the incident beam, the probability of the interactions between an element of the compound with photons will change accordingly. Regarding the dependency of types of interactions on the type of the element and energy of the photon, there is a possibility that in different energies, the atomic number also changes. Numerous researchers have studied effective atomic numbers for photons^7,13–16^. Meanwhile, quite a few have discussed Z_eff_ for electron, alpha, proton, and carbon in different energies^1,13,17^. Kurudirek calculated the effective atomic number for some charged particles using the interpolation method of stopping powers^1,3^.

In the present work, Z_eff_ of 19 materials relevant to dosimetry is calculated for total electron interactions in the wide kinetic energy range of 10 keV to 1 GeV. Because of the dependency of stopping power on some experimental parameters, in this paper, a direct method is used to calculate the effective atomic number. In this method, interpolations were not used, and for each compound, the effective atomic number can be calculated directly by its stopping power and average ionization and excitation energy.

The variation of Z_eff_ through the entire kinetic energy region is also investigated. Moreover, the water, as well as tissue equivalence properties of the materials, is studied in the entire kinetic energy region. The effective atomic number was calculated for 19 materials equivalent to tissue, and dosimetry compounds used Bethe's formula^18^.

The determinations are done utilizing collision-stopping power. The kinetic energy region of 10 keV–20 MeV is considered for electrons, and the proton particles are studied in the energy kinetic energy range of 60 MeV to 200 MeV. Furthermore, Z_eff_ calculated for the alpha particles with a kinetic energy of 9 MeV and carbon ions of 2 GeV.

## Methods

Due to the significant importance of determining the body tissue's effective atomic number for charged particles, an attempt is made to study 19 tissue equivalent materials and dosimetry compounds, as shown in Table 1. Coulomb interaction was considered, and the Z_eff_ was calculated in different kinetic energy ranges for electron, proton, alpha, and carbon ions. The effective atomic number for the mentioned particles was determined using the collisional stopping power equation and the stopping powers extracted from the NIST library. It must be indicated that, since the atomic number is not specified for compounds, Bethe's formula cannot be used directly for determining stopping power. As a result, the effective atomic number was determined based on the stopping power value and an unknown atomic number.

**Table 1:** Chosen compounds and their applications.

	Compound	Application		Compound	Application
1	Water	Tissue equivalent	11	Aluminum oxide	Dosimeter
2	Beryllium oxide	Dosimeter	12	Lithium fluoride	Dosimeter
3	Calcium fluoride	Dosimeter	13	Calcium sulfate	Dosimeter
4	Alanine	Dosimeter	14	Lithium tetra borate	Dosimeter
5	Polyethylene	Phantom construction	15	Cadmium telluride	Dosimeter and detector
6	Magnesium oxide	Scintillation detector	16	Cesium iodine	Detector
7	Cadmium tungstate	Detector	17	Polycarbonate	Detector and Phantom construction
8	Teflon	Phantom construction as bone equivalent material	18	Perspex	Dosimeter and Phantom construction as tissue equivalent material
9	Bismuth germanium oxide	Detector	19	Polystyrene	Phantom construction as blood-equivalent material
10	Nylon type 6 and 6/6	Phantom construction as brain-equivalent material			

Assuming that all the atoms and their electrons behave independently from each other and all the kinetic energy only will be spent on ionization and excitation, in that case, the collisional stopping power of the electrons is calculated by Bethe's formula:

1 $$-\frac{dE}{dx} \left(\frac{MeV}{m}\right)=4\pi {{r}_{0}}^{2}{z}^{2}\frac{m{c}^{2}}{{\beta }^{2}} NZ\left\{\mathit{ln}\left(\frac{\beta \gamma \sqrt{\gamma -1}}{I}m {c}^{2}\right)+\frac{1}{2{\gamma }^{2}}\left[(\frac{{\left(\gamma -1\right)}^{2}}{8}+1-\left({\gamma }^{2}+2\gamma -1\right)ln2)\right]\right\}$$

where $4\pi {{r}_{0}}^{2}={10}^{-24}{\mathrm{cm}}^{2}$, ${r}_{0}=\frac{{e}^{2}}{m{c}^{2}}=2.818*{10}^{-15}\mathrm{m}$, $m{c}^{2}=0.511 \mathrm{MeV}$, $m$ is the rest mass of an electron, $Z$ is the atomic number of the medium, $z$ is the particle charge, $N$ is the atomic mass, $I$ is the average ionization and excitation kinetic energy, $\gamma = \frac{1}{\sqrt{1-{\beta }^{2}}}$ and $\beta =\frac{v}{c }$, $c=3*{10}^{8} \mathrm{m}/\mathrm{s}$.

Also, the collisional stopping power of ionization and excitation for heavy ions like $\alpha$, $t$, $d$, and $p$ is equal to:

2 $$-\frac{dE}{dx}(MeV/m)=4\pi {{r}_{0}}^{2}{z}^{2}\frac{m{c}^{2}}{{\beta }^{2}}NZ\left[\mathrm{ln}\left(\frac{2m{c}^{2}}{I}{\beta }^{2}{\gamma }^{2}\right)-{\beta }^{2}\right]$$

Since there are some assumptions in Bethe's formula, in high kinetic energy ranges, the values of stopping power calculated using this relation may differ from experimental data. Although the basis of ESTAR code calculations is Bethe's formula, it uses some experimental parameters such as density effect correction. So, this code's calculated values are closer to the experimental values. Therefore, the collisional stopping powers were calculated for different elements to select the appropriate kinetic energy range, utilizing Eq. (1). These values were compared with stopping powers gathered from the ESTAR database^19^. The valid kinetic energy range is selected as the range that relative difference between the calculated stopping power using Bethe's formula and the stopping power extracted from the NIST data library is less than 20%.

In pursuance of determining the effective atomic number by means of the collisional stopping power of the electrons, the values of ${\left(\frac{dE}{dx}\right)}$, were gathered from the ESTAR database, and the values for Z_eff_ were determined using Eq. (3).

3 $$Z=\left(\frac{\left|\frac{dE}{dx}\right|}{ 4\pi {{r}_{0}}^{2}{z}^{2}\frac{m{c}^{2}}{{\beta }^{2}} N\left\{\mathit{ln}\left(\frac{\beta \gamma \sqrt{\gamma -1}}{I}m {c}^{2}\right)+\frac{1}{2{\gamma }^{2}}\left[(\frac{{\left(\gamma -1\right)}^{2}}{8}+1-\left({\gamma }^{2}+2\gamma -1\right)ln2)\right]\right\}}\right)$$

Also, to calculate the collisional stopping power for proton, alpha, and carbon, the same procedures were taken. As mentioned before, the collisional stopping power of the elements of each compound was obtained by Eq. (2).

Moreover, the data was extracted from ASTAR^20^, PSTAR^21^, and SRIM databases^22^. Final data were compared to each other, so those ranges of kinetic energy where the relative error of the results were less than 20% were introduced as valid kinetic energy range. Also, the values for Z_eff_ were determined using Eq. (4).

4 $$Z=\left(\frac{\left|\frac{dE}{dx}\right| }{ 4\pi {{r}_{0}}^{2}{z}^{2}\frac{m{c}^{2}}{{\beta }^{2}}NZ\left[\mathit{ln}\left(\frac{2m{c}^{2}}{I}{\beta }^{2}{\gamma }^{2}\right)-{\beta }^{2}\right]}\right)$$

## Results

### Z_eff_ of electron

The data from the ESTAR database and theoretical data of the collisional stopping power of electrons were determined and compared. The kinetic energy ranges where the percentage of the relative difference of the results is less than 20% are identified as valid kinetic energy ranges. The compounds with such features are set in Table 2. As can be seen, for the electron interaction, the percentage of the relative difference in low kinetic energy ranges is less than 20%.

**Table 2:** Valid kinetic energy range for electron interactions.

	Compound	Kinetic energy range (MeV)		Compound	Kinetic energy range (MeV)
1	Water	0.01–20	11	Aluminum oxide	0.01–20
2	Beryllium oxide	0.01–15	12	Lithium fluoride	0.01–20
3	Calcium fluoride	0.01–30	13	Calcium sulfate	0.01–40
4	Alanine	0.01–20	14	Lithium tetraborate	0.01–20
5	Polyethylene	0.01–20	15	Cadmium telluride	0.01–70
6	Magnesium oxide	0.01–20	16	Cesium iodine	0.01–90
7	Cadmium tungstate	0.01–40	17	Polycarbonate	0.01–20
8	Teflon	0.01–20	18	Perspex	0.01–20
9	Bismuth germanium oxide	0.01–60	19	Polystyrene	0.01–20
10	Nylon type 6 and 6/6	0.01–20			

According to Table 3 data, the effective atomic numbers for different compounds used as dosimeters or detectors are calculated for several specific energies in valid kinetic energy ranges. Since Eq. (1) is most important for electron interactions in low energies, the main focus is on these kinetic energy ranges.

**Table 3:** Results of effective atomic number for different compounds in a few of the kinetic energy using collisional stopping power of electron. The uncertainties are due to the uncertainty of the stopping powers in the NIST data library.

	Compound	Effective atomic number (*Z*_eff_)					Total Number of electrons
		Electron kinetic energy (MeV)					
		0.01	0.1	1	10	20	
1	Water	9.98 ± 0.20	9.98 ± 0.20	9.85 ± 0.20	8.83 ± 0.18	8.52 ± 0.17	10
2	Beryllium oxide	12.0 ± 0.2	12.0 ± 0.2	12.0 ± 0.2	10.0 ± 0.2	10.0 ± 0.2	12
3	Calcium fluoride	38.0 ± 0.8	38.0 ± 0.8	38.0 ± 0.8	33.9 ± 0.7	32.7 ± 0.6	38
4	Alanine	47.9 ± 1.0	47.9 ± 1.0	46.7 ± 1.0	41.9 ± 0.8	40.4 ± 0.8	48
5	Polyethylene	16.0 ± 0.3	16.0 ± 0.3	16.0 ± 0.3	14.0 ± 0.3	13.5 ± 0.3	16
6	Magnesium oxide	20.0 ± 0.4	20.0 ± 0.4	19.6 ± 0.4	17.6 ± 0.3	17.0 ± 0.3	20
7	Cadmium tungstate	154 ± 3	154 ± 3	150 ± 3	139 ± 3	135 ± 3	154
8	Teflon	47.9 ± 1.0	47.9 ± 1.0	47.0 ± 1.0	42.2 ± 0.8	40.7 ± 0.8	48
9	Bismuth germanium oxide	523 ± 10	523 ± 10	515 ± 10	479 ± 10	465 ± 10	524
10	Nylon type 6 and 6/6	61.9 ± 1.2	61.9 ± 1.2	60.3 ± 1.2	54.2 ± 1.1	52.2 ± 1.0	62
11	Aluminum oxide	49.9 ± 1.0	49.9 ± 1.0	48.8 ± 1.0	44.0 ± 0.9	42.4 ± 0.8	50
12	Lithium fluoride	12.0 ± 0.2	12.0 ± 0.2	11.7 ± 0.2	10.5 ± 0.2	10.1 ± 0.2	12
13	Calcium sulfate	67.8 ± 1.3	67.7 ± 1.4	66.4 ± 1.3	60.4 ± 1.2	58.3 ± 1.2	68
14	Lithium tetra borate	81.8 ± 1.6	81.8 ± 1.6	79.8 ± 1.6	71.7 ± 1.4	69.0 ± 1.4	82
15	Cadmium telluride	99.8 ± 2.0	99.8 ± 2.0	98.4 ± 2.0	92.5 ± 1.8	89.8 ± 1.8	100
16	Cesium iodine	107 ± 2	107 ± 2	107 ± 2	101 ± 2	98.2 ± 2.0	108
17	Polycarbonate	153 ± 3	153 ± 3	150 ± 3	135 ± 3	130 ± 3	154
18	Perspex	53.9 ± 1.1	53.9 ± 1.1	52.9 ± 1.1	47.5 ± 1.0	45.8 ± 1.0	54
19	Polystyrene	55.9 ± 1.1	55.9 ± 1.1	54.8 ± 1.1	49.4 ± 1.0	47.6 ± 1.0	56

As it is apparent, in low kinetic energy ranges, the effective atomic number is nearly equal to the total number of electrons owned by the compound. These results can be explained since they rely on collisional stopping power. While an electron transports a medium, it will have Coulomb interaction with all the electrons. As the interaction happens for all the electrons, the value of the effective atomic number would be identical to the number of electrons. In other words, the electron passes through a medium that includes the electrons of all the elements without considering their weight percentages. By increasing the kinetic energy, the electron's movement through the medium is also enhanced; Thus, less Coulomb forces affect the electron, and as a consequence, the effective atomic number decreases.

It can be observed that Z_eff_ for some compounds like beryllium Oxide and lithium fluoride, which are known as tissue equivalent dosimeters, are close in value. Also, the relative difference between effective atomic number of the alanine and polystyrene, known as a blood equivalent tissues, is 14%. Teflon, Perspex, Nylon, and even Polycarbonate, widely used as bone, tissue, and brain equivalent material, have slightly different atomic numbers compared with bone, tissue, and brain. Nevertheless, studies on some widely known detector materials, namely, Cadmium telluride, Cadmium tungstate, and Bismuth germanium oxide, resulted from high effective atomic numbers, which make them suitable to be utilized as detectors.

**Figure 1:**
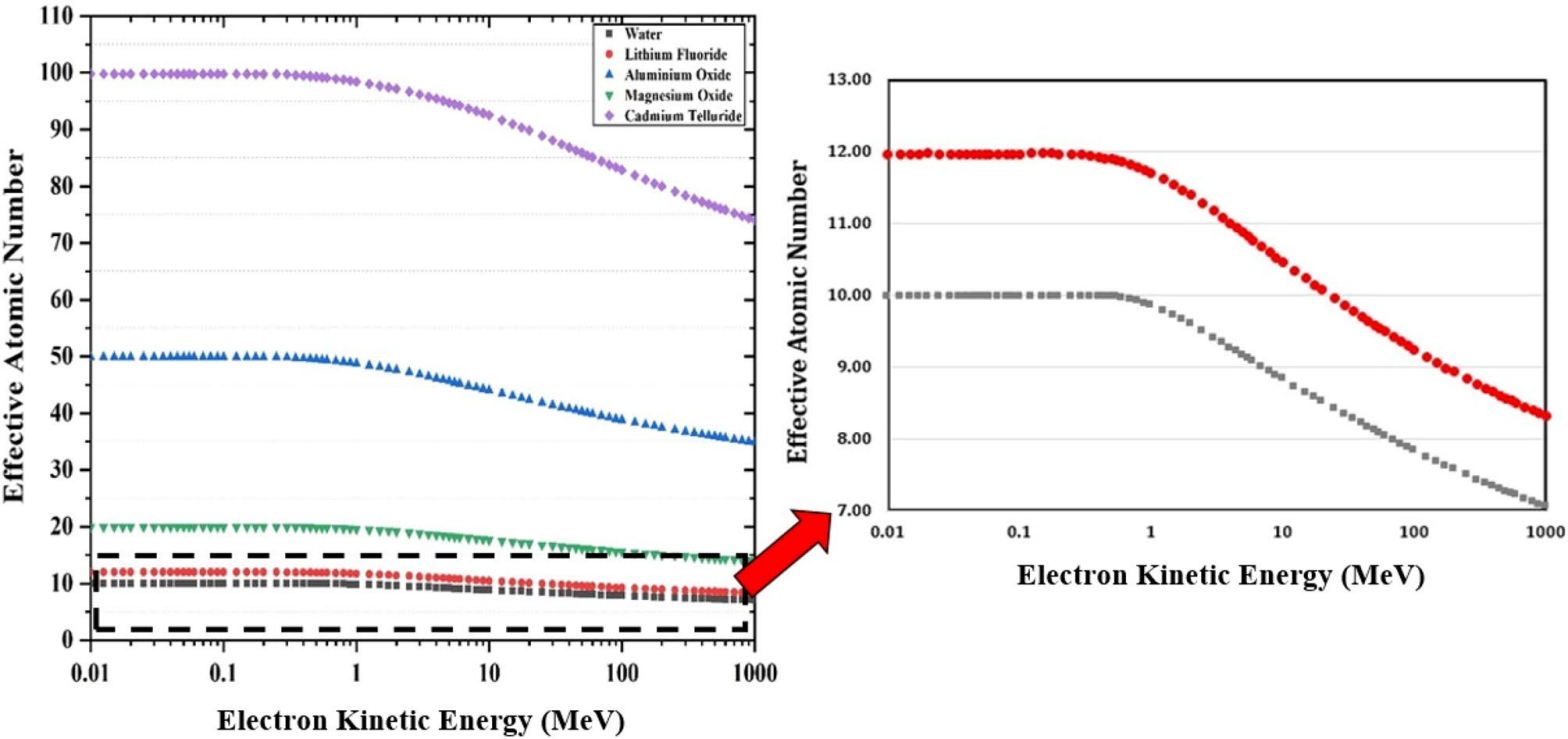
Variation of Z_eff_ with kinetic energy of Electron for water, lithium fluoride, aluminum oxide, magnesium oxide and, cadmium telluride. At low energies the effective atomic number is close to the number of electrons in molecular unit of compound.

According to Table 3 data, it can be obtained that the effective atomic number values decrease by increasing the kinetic energy of the electron particles. The variations in effective atomic number by increasing kinetic energy of the electron for water, lithium fluoride, aluminum oxide, magnesium oxide, and cadmium telluride are depicted in Fig. 1.

### Z_eff_ of the proton, alpha, and carbon particles

The experimental data from SRIM software, ASTAR, and PSTAR database and theoretical data of collisional the stopping power of electron have been determined and compared with each other. The kinetic energy ranges where the relative difference percentages of the results are less than 20% are identified as valid kinetic energy ranges (Table 4).

**Table 4:** Valid kinetic energy range for proton, alpha, and carbon interactions.

	Compound	Proton kinetic energy range (MeV)	Alpha kinetic energy range (MeV)	Carbon kinetic energy range (MeV)
1	Water	0.05–1000	1.5–1000	20–1000
2	Beryllium oxide	0.06–1000	1.5–1000	30–1000
3	Calcium fluoride	0.2–1000	0.5–1000	25–1000
4	Alanine	1.5–1000	3.5–1000	50–1000
5	Polyethylene	0.04–1000	0.8–1000	15–1000
6	Magnesium oxide	0.2–1000	0.7–1000	30–1000
7	Cadmium tungstate	0.6–1000	2.5–1000	70–1000
8	Teflon	0.08–1000	1.5–1000	20–1000
9	Bismuth germanium oxide	0.6–1000	100–1000	40–1000
10	Nylon type 6 and 6/6	0.05–1000	1–1000	20–1000
11	Aluminum oxide	0.1–1000	1.5–1000	30–1000
12	Lithium fluoride	0.06–1000	1.5–1000	30–1000
13	Calcium sulfate	0.2–1000	0.4–1000	30–1000
14	Lithium tetra borate	0.08–1000	1–1000	30–1000
15	Cadmium telluride	0.6–1000	2.5–1000	30–1000
16	Cesium iodine	0.6–1000	2–1000	40–1000
17	Polycarbonate	0.05–1000	1.5–1000	20–1000
18	Perspex	0.05–1000	1–1000	50–1000
19	Polystyrene	0.05–1000	1–1000	20–1000

In therapeutic processes, 60 MeV up to 250 MeV proton, 5 MeV up to 9 MeV alpha, and 1200 MeV up to 2400 MeV carbon are usually used. Therefore, determining Z_eff_ in the mentioned energies is more critical.

**Figure 2:**
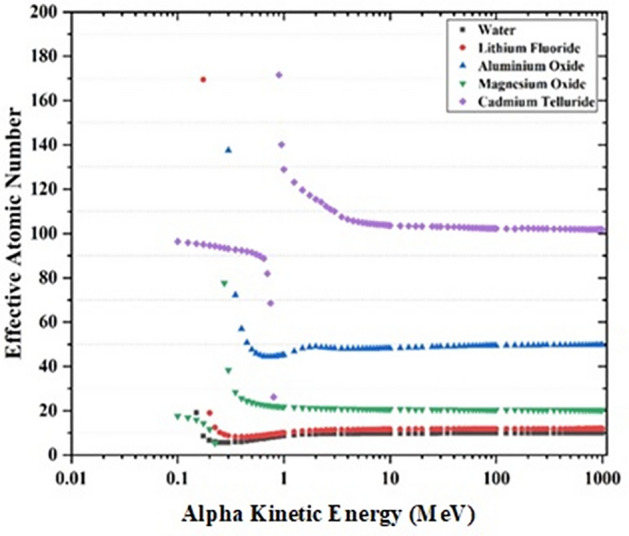
Variation of Z_eff_ with kinetic energy of Alpha for water, lithium fluoride, aluminum oxide, magnesium oxide and cadmium telluride. At low energies the effective atomic number is close to the number of electrons in molecular unit of compound.

**Figure 3:**
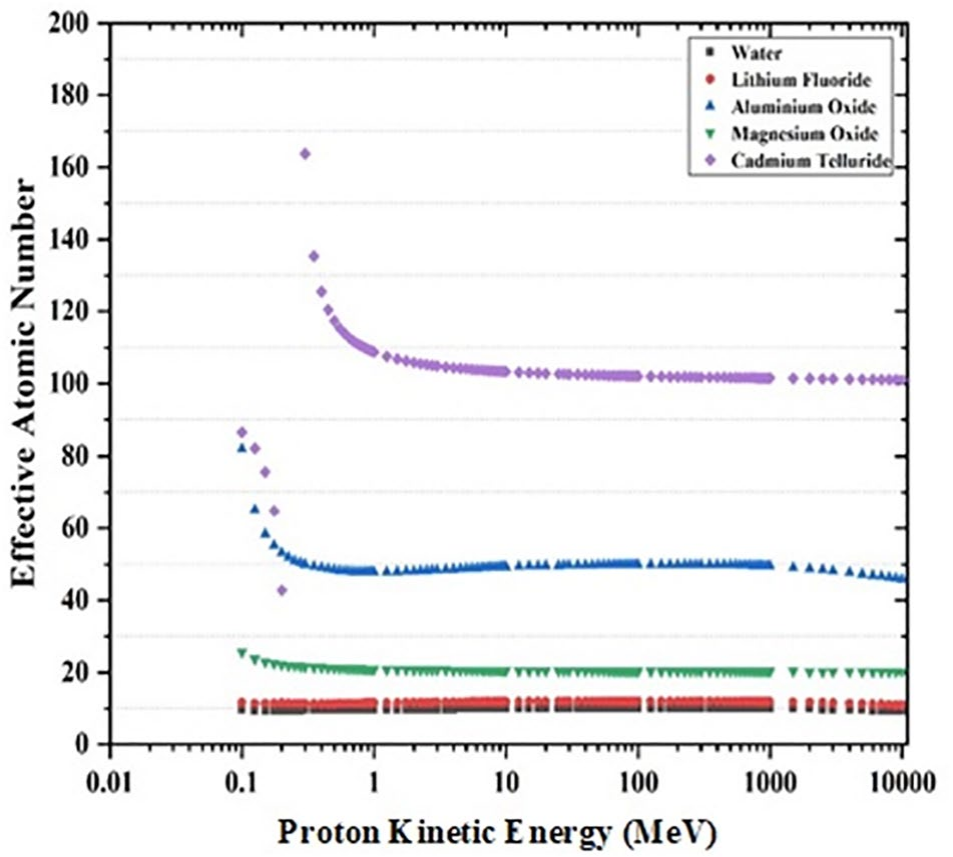
Variation of Z_eff_ with kinetic energy of Proton for water, lithium fluoride, aluminum oxide, magnesium oxide and cadmium telluride. At low energies the effective atomic number is close to the number of electrons in molecular unit of compound.

**Figure 4:**
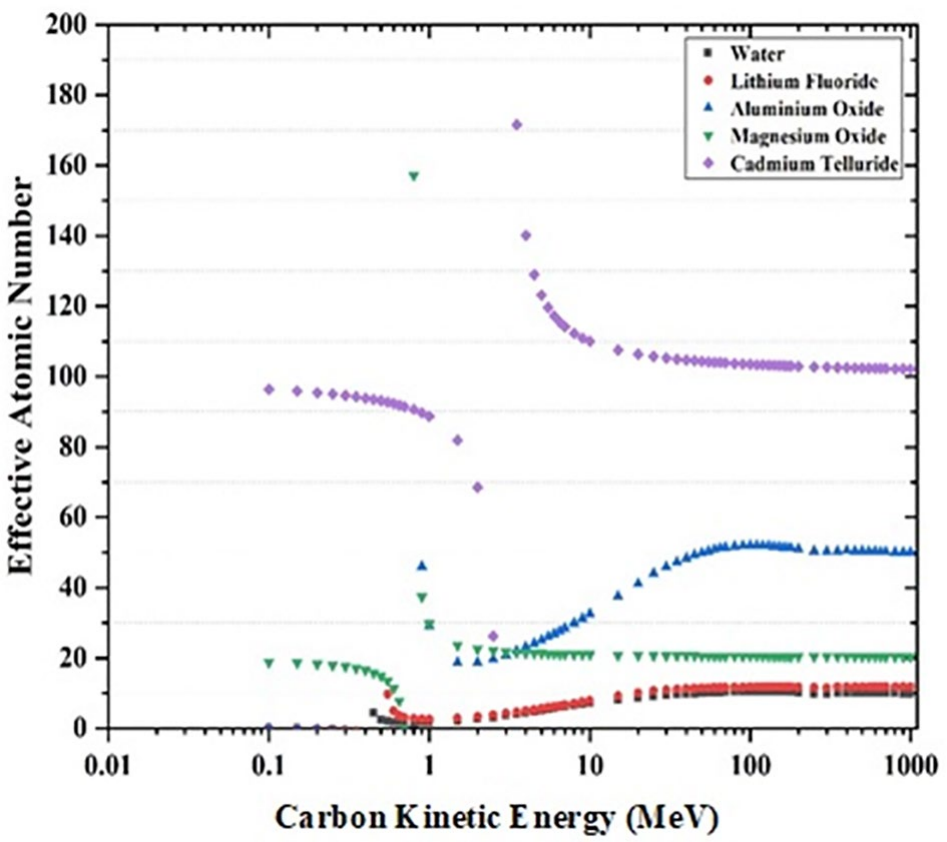
Variation of Z_eff_ with kinetic energy of Carbon for water, lithium fluoride, aluminum oxide, magnesium oxide, and cadmium telluride. At low energies the effective atomic number is close to the number of electrons in molecular unit of compound.

The results of calculating the effective atomic number for all the compounds in the energies mentioned above ranges of the proton, alpha, and carbon particles are shown in Table 5. Since Eq. (2) is most important in high kinetic energy ranges, this area's data is selected and studied. As can be seen, for the proton and heavy charged particles interactions, the Z_eff_ value, is nearly equal to the total number of electrons of the compound. The results can be explained as the determinations are based on collisional stopping power. Variations of Z_eff_ with the kinetic energy of alpha, proton and, carbon for water, lithium fluoride, aluminum oxide, magnesium oxide and, cadmium telluride are shown in Figs. 2, 3, 4.

**Table 5:** Results of effective atomic number in a few of kinetic energies for different compounds using collisional stopping power of proton, alpha, and carbon. The uncertainties are due to the uncertainty of the stopping powers in the SRIM code.

	Compound	Effective Atomic number (*Z*_eff_)						Total number of electrons
		Proton kinetic energy (MeV)				Alpha kinetic energy (MeV)	Carbon kinetic energy (MeV)	
		60	100	150	200	9	2000	
1	Water	9.97 ± 0.45	9.96 ± 0.45	9.97 ± 0.45	9.97 ± 0.45	9.74 ± 0.43	9.86* ± 0.54	10
2	Beryllium oxide	12.3 ± 0.5	12.3 ± 0.5	12.2 ± 0.5	12.2 ± 0.5	12.3 ± 0.5	12.0 ± 0.5	12
3	Calcium fluoride	37.8 ± 1.7	37.8 ± 1.7	37.9 ± 1.7	37.9 ± 1.7	36.8 ± 1.7	37.7 ± 1.9	38
4	Alanine	43.8 ± 2.0	43.8 ± 2.0	43.8 ± 2.0	43.8 ± 2.0	42.8 ± 1.9	42.9 ± 2.4	48
5	Polyethylene	16.00 ± 0.7	16.00 ± 0.7	16.00 ± 0.7	16.00 ± 0.7	15.7 ± 0.7	15.7 ± 0.9	16
6	Magnesium oxide	20.1 ± 0.9	20.1 ± 0.9	20.1 ± 0.9	20.1 ± 0.9	20.0 ± 0.9	19.7 ± 1.1	20
7	Cadmium tungstate	139 ± 6	140 ± 6	141 ± 6	141 ± 6	123 ± 5	135 ± 7	154
8	Teflon	47.8 ± 2.2	47.9 ± 2.2	47.9 ± 2.2	47.9 ± 2.2	46.5 ± 2.2	46.6 ± 2.6	48
9	Bismuth germanium oxide	528 ± 24	528 ± 24	530 ± 24	531 ± 24	410 ± 18	462 ± 25	524
10	Nylon type 6 and 6/6	61.8 ± 3	61.8 ± 3	61.8 ± 3	61.8 ± 3	60.5 ± 2.7	60.7 ± 3.3	62
11	Aluminum oxide	49.8 ± 2.2	49.8 ± 2.2	49.8 ± 2.2	49.9 ± 2.2	48.4 ± 2.2	49.6 ± 2.7	50
12	Lithium fluoride	12.0 ± 0.5	12.0 ± 0.5	12.0 ± 0.5	12.0 ± 0.5	11.7 ± 0.5	11.9 ± 0.6	12
13	Calcium sulfate	67.5 ± 3.0	67.5 ± 3.0	67.5 ± 3.0	67.5 ± 3.0	66.1 ± 3.0	66.0 ± 3.6	68
14	Lithium tetra borate	81.7 ± 3.7	81.8 ± 3.7	81.8 ± 3.7	81.8 ± 3.7	79.7 ± 3.6	81.2 ± 4.5	82
15	Cadmium telluride	102 ± 5	102 ± 5	102 ± 5	102 ± 5	98.2 ± 4.4	99.4 ± 6	100
16	Cesium iodine	105.90 ± 4.76	106.50 ± 4.79	106.85 ± 4.80	107.03 ± 4.81	101.19 ± 4.55	107.56 ± 5.92	108
17	Polycarbonate	153 ± 7	153 ± 7	153 ± 7	153 ± 7	149 ± 7	150 ± 8	154
18	Perspex	53.8 ± 2.4	53.9 ± 2.4	53.9 ± 2.4	53.9 ± 2.4	52.6 ± 2.4	48.9 ± 2.7	54
19	Polystyrene	55.8 ± 2.5	55.8 ± 2.5	55.7 ± 2.5	55.7 ± 2.5	54.6 ± 2.5	55.1 ± 3.0	56

*The effective atomic number of water for carbon ions with kinetic energy of 4.6 GeV is calculated 9.81. It should be mentioned that 4.6 GeV carbons and 200 MeV protons have the same range in water.

When a charged particle passes an absorber, it loses kinetic energy through several events. The particle interacts with many electrons; moreover, in low energies, a positively charged particle intends to gather the electrons of the absorber. This process causes a decrease in the particle charge and lowers the loss of linear kinetic energy. At the end of the path, the particle absorbs Z electrons and turns into a neutral atom.

Results showed that in low energies of the electron, the effective atomic number is close to the number of electrons in each compound molecule. As the results are obtained using collision-stopping power, it can be concluded that the electron has had Coulomb interaction with all the electrons while passing the medium. So, the value of Z_eff_ would be equal to the total number of electrons. In other words, the electron has entered a medium containing all elements' electrons regardless of their weight percentage. The effective atomic number will decrease by increasing kinetic energy since the electron will move faster in the medium and affect less Coulomb forces.

Accordingly, Z_eff_ does not have a unique value to be used in the entire kinetic energy region of certain ionizing radiation due to the fact that multi-element materials have many constituents with different atomic numbers, which results in different radiation interaction probabilities in different kinetic energy regions. Therefore, Z_eff_ is considered as a kinetic energy-dependent parameter that depends on the chemical composition of the corresponding material. In these circumstances, some materials that are equivalent in the presence of photons might not be equivalent in the presence of electrons, protons, alpha, and carbon. As in some previous studies, the kinetic energy of photons has been indicated as an effective parameter assessment of two equivalent materials.

In the present work, the collision-stopping power method is used to calculate the effective atomic number in different energies. Studies showed that an effective atomic number depends on the particle's kinetic energy, in which for charged particles, the interpolation of collision-stopping power values based on the element's atomic number is considered. Although the basis of final results in ESTAR, PSTAR, ASTAR, and SRIM is, in fact, Bethe's formula. But it should be considered that in the calculations of library data, ionization and excitation mean kinetic energy parameters, layers correction, and density effect correction also have been applied. Regarding the process of transferring the kinetic energy of charged particles to the medium, the calculated effective atomic number is equal to the total electrons of each compound, and its value will decrease by increasing kinetic energy.

## Conclusion

In this paper, an effort has been paid to calculate the effective atomic number for some of the charged particles using a simple direct method. The effective atomic number of some materials used in dosimetry and some tissue equivalent for electron, proton, alpha, and carbon interactions were calculated. The results of these calculations are equal to the total electrons in each molecule of the compounds, which is justifiable by Bethe's formula physics. Consequently, some materials considered to be equivalent were proved not to be quite equivalent. These incompatibilities cannot reject or approve the commonly used methods for determining the Z_eff_, but in fact, they can result in a discussion on valid kinetic energy ranges for each method. In other words, regarding the particles' type, incident kinetic energy, and different kinetic energy ranges, one of the mentioned methods would be valid for determining the effective atomic number.

## Data Availability

All data generated or analyzed during this study are included in this published article.
